# Involvement of the Ipsilateral Tongue, an Intraoral Structure of Referred Pain due to Entrapment of the Greater Occipital Nerve

**DOI:** 10.1155/crnm/3993982

**Published:** 2024-12-19

**Authors:** Byung-chul Son

**Affiliations:** ^1^Department of Neurosurgery, Seoul St. Mary's Hospital, College of Medicine, The Catholic University of Korea, Seoul, Republic of Korea; ^2^Catholic Neuroscience Institute, College of Medicine, The Catholic University of Korea, Seoul, Republic of Korea

**Keywords:** entrapment neuropathy, greater occipital nerve, referred pain, tongue, trigeminal nucleus, trigemiocervical complex

## Abstract

This study reports a rare case of referred pain in the trigeminal nerve distribution caused by entrapment of the greater occipital nerve (GON). Notably, the pain extended to the ipsilateral tongue, an unusual intraoral involvement. GON entrapment can lead to sensitization in secondary nociceptive neurons within the trigeminocervical complex (TCC), which receives signals from both trigeminal and occipital nerves, causing referred facial pain. A 55-year-old female presented with chronic left temporo-occipital pain, along with pain in her left periorbital area, ear canal, gum, and a 20-year history of atypical facial pain on her left tongue and lower lip. Following GON decompression, her temporo-occipital pain and facial symptoms improved, with a significant reduction in burning pain on her tongue and resolution of lip tingling. The TCC, comprising convergent inputs from trigeminal and occipital nerves, is the anatomical basis of referred craniofacial pain. Chronic GON entrapment can sensitize second-order neurons in the TCC and medullary dorsal horn, explaining this unusual referred pain to the intraoral structures.

## 1. Introduction

Patients with primary headaches often experience pain in both the frontal head, which is innervated by the trigeminal nerve and the occipital region, which is innervated by the greater occipital nerve (GON) [1]. In addition, when structures in the posterior head and neck such as the infratentorial dura mater, cervical roots, tumors in the posterior fossa, and subcutaneous tissue innervated by upper cervical roots in humans innervated by the GON are stimulated, it can be perceived as frontal pain [1–4]. The anatomical basis of this pain referral between the frontal trigeminal and high cervical area, innervated by the GON, is due to the convergence of nociceptive afferents from the high cervical region and the trigeminal system. This occurs through the second-order nociceptive neurons in the dorsal horn of the high cervical cord, specifically from the C2 segment up to the medullary dorsal horn (MDH) in the caudal trigeminal nucleus [1, 5, 6]. The “trigeminocervical complex (TCC)” refers to the caudal spinal trigeminal nucleus and laminae I-II of the upper two cervical spinal segments, as well as the commissural division of the nucleus of the solitary tract and its caudal extension into spinal lamina X [7]. These second-order trigeminal nociceptive neurons in the TCC are sensitive to the dura and receive input from other trigeminal afferents, such as the facial and corneal areas [8]. Convergence of these inputs, along with central sensitization of the second-order nociceptive neurons, provides a physiological explanation for the clinical phenomenon of spread and referred pain between the trigeminal and cervical areas. This means that pain originating from one area can be felt as if it is coming from a different area [1, 5, 7–11].

In recent years, there have been ongoing reports suggesting that the chronic noxious afferent input caused by the entrapment of the GON can lead to the sensitization and hypersensitivity of secondary neurons in the TCC. This, in turn, can result in referred pain in the facial trigeminal nerve distribution [12–20]. Referral to the orofacial area occurs not only in the V1 (ophthalmic) region but also in V2 (maxillary) and V3 (mandibular) regions. It even causes hemifacial sensory changes [13]. Referred pain from GON entrapment has also been reported to cause deep ear pain [16]. The nerves responsible for the sensory innervation of the external auditory canal and middle ear include the auriculotemporal branch of the mandibular nerve (V3), the facial nerve, the glossopharyngeal nerve, and the vagal nerve [21, 22]. All these four nerves, known for carrying pain signals to the craniofacial structures, end in the caudal trigeminal nucleus [23]. When the TCC becomes hypersensitive due to GON entrapment, the resulting referred pain can extend beyond the facial trigeminal distribution area to include the arm and leg on the same side, going beyond the craniocervical region [18]. Furthermore, the referred pain from the extra-trigeminal region can extend beyond the affected limb and affect the entire body [19]. This widespread extension of referred pain, resulting from the entrapment of the GON, is thought to be caused by sensitization of the third-order nociceptive neurons in the thalamus [19, 24, 25]. Another possible explanation for this phenomenon is that spontaneous pain may occur in various parts of the body as a result of dysfunction in the descending pain-modulating pathway of the brain stem. This dysfunction may be caused by sensitization and hyperexcitation of the medullary dorsal horn and trigeminal brainstem sensory nuclear complex (TBSNC) [26, 27]. It has been observed that referred facial trigeminal pain, which is caused by entrapment of the GON, can be alleviated by decompression of the GON [12–20].

This report presents a case of chronic GON entrapment leading to referred pain in the tongue and lips. It is worth noting that the mandibular division of the trigeminal nerve covers these areas. Previous studies have documented referred pain in the eyes, jaw, and ears, but this is the first report to indicate that the pain can extend to the tongue, an intraoral structure. Furthermore, the patient has been experiencing pain on one side of the tongue for over 20 years, predating the onset of occipital pain. As a result, this unexplained tongue pain, which lacks an identified structural cause, has been classified as atypical facial pain.

## 2. Case Report

**Figure 1:**
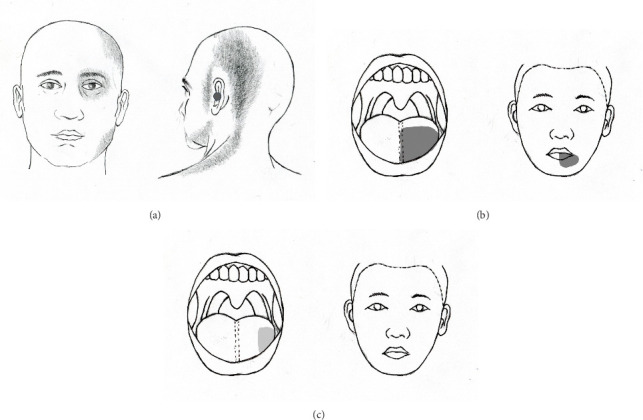
Schematic drawing showing the distribution of craniofacial pain caused by greater occipital nerve (GON) entrapment. (a) The location (gray areas) of temporal–occipital pain along with left periorbital, preauricular, and mental pain. It has also extended to the nape of the neck and shoulders. It was accompanied by dull pain such as a bruise in the left ear canal (black circle). (b) Distribution (gray areas) of left tongue and lip pain diagnosed as atypical facial pain for 20 years. (c) Drawing showing improvement and reduction in the extent of left tongue and lip pain.

A 55-year-old female patient presented with a chronic headache localized to the left temporo-occipital region. In conjunction with this headache, she experienced pain in the left facial region. The onset of pain was sudden, occurring after the patient underwent emotional stress 14 months prior to seeking treatment. She described the pain as throbbing in the left temporo-occipital area, accompanied by a sensation around her left eye, which felt as though it might “fall out.” Within a few days, she developed aching and tightening pain in the suboccipital and lateral aspects of her neck. In addition, she reported pain in the left preauricular cheek area and a deep, uncomfortable, bruise-like pain in her left ear (Figure 1(a)). The patient also experienced intermittent high-pitched tinnitus that exacerbated with the pain in her left ear. Moreover, she reported constant dull pain in her left jaw's gums. The persistent headache, facial pain, and neck discomfort were present throughout the day. Notably, these symptoms were not exacerbated by specific activities and remained continuous.

The patient underwent comprehensive evaluations in ophthalmology, dentistry, and otolaryngology, but all tests, including CT scans of the sinuses and temporal bone, were unremarkable. Three neurologists diagnosed her with migraines. MRI and CT scans of the brain also revealed no abnormalities. Despite being prescribed over five anti-migraine medications, including triptans and nonsteroidal anti-inflammatory drugs for several months, none provided relief. In addition, she received two botulinum toxin injections and two calcitonin gene-related peptide (CGRP) antagonist injections, which were similarly ineffective. Subsequently, she was evaluated by an orthopedic surgeon and a neurosurgeon for the left suboccipital headache and lateral neck pain, but no significant abnormalities were found on examination or MRI of the cervical spine. Eventually, the patient was referred to this author due to uncontrolled pain while undergoing physical therapy and acupuncture treatment, despite taking six medications, including triptans, pregabalin, NSAIDs, muscle relaxants, antidepressants, and Mypol.

Concurrently, the patient had experienced chronic pain in her left tongue and lips for the past 20 years. This condition began suddenly when she first noted pain in the left half of her tongue, the medial aspect of her left lower lip, and the left neck. She described a persistent bitter and burning sensation on the left side of her tongue, along with recurring tingling pain in her lower left lip (Figure 1(b)). Notably, there was no pain in the remainder of her mouth. During this time, she suffered from severe neck pain that limited her ability to turn her head for several months. While the pain in her lips was intermittent, the pain in her tongue was constant and not affected by activities such as eating, brushing teeth, or yawning. The patient sought treatment from multiple specialists, including dentistry, neurology, neurosurgery, and otolaryngology, yet no underlying cause was identified. Approximately 6 months later, her neck pain gradually improved; however, the pain in her tongue and lips persisted. One year following the onset of her symptoms, she was diagnosed with trigeminal neuralgia at a pain clinic. Despite undergoing a trigeminal nerve block with alcohol, she did not experience relief.

Ultimately, she was diagnosed with atypical facial pain. Over several years, she sought treatment from various dental and neurology clinics, receiving a combination of anticonvulsants (including gabapentin and pregabalin), clonazepam, and antidepressants. After 5 years of pharmacotherapy, she opted to discontinue treatment for her atypical facial pain and manage it independently. She rated the intensity of her pain on an 11-point numerical rating scale (NRS-11) as 3 to 4. Remarkably, the pain in her left tongue and lips remained stable, despite the emergence of the left temporo-occipital headache and left facial pain 14 months ago.

Upon examination, no objective sensory changes were noted in the patient's face, despite her ongoing pain and discomfort in that region and her neck. No neurological abnormalities were detected in the masticatory function of the trigeminal nerve, other cranial nerves, or extremities. Furthermore, no allodynia or tenderness was found in the occipital, neck, or facial regions nor were there movement restrictions in her neck or jaw. Laboratory evaluations, including erythrocyte sedimentation rate (ESR) and uric acid, returned normal results. An MRI performed to investigate potential trigeminal neuropathy did not reveal any vascular compression or abnormal enhancement of the trigeminal nerve. In addition, a CT scan of the cervical spine was conducted to assess for structural lesions in the craniovertebral junction and along the pathway from the C2 nerve root to the GON, but no abnormalities were detected. Importantly, the patient had no significant medical history, including hypertension or diabetes, apart from chronic intermittent neck and shoulder pain.

Given the possibility of referred trigeminal facial pain due to GON entrapment, an occipital nerve block (ONB) was performed on the left GON using 2 mL of 2% lidocaine. This intervention resulted in an 80% reduction in the intensity of pain in the temporo-occipital, suboccipital, periorbital, and malar regions, lasting for 6–8 h. However, the chronic tongue pain did not improve with the ONB, although relief in the left lower lip lasted approximately 3 h. Six hours after the ONB, the pain in the facial, occipital, and neck regions returned to baseline intensity. A second ONB of the left GON was performed 2 weeks later, yielding a similar temporary improvement. Considering the chronicity and resistance of the pain, as well as the potential for referred facial pain due to chronic GON entrapment, we recommended GON decompression after obtaining written informed consent.

**Figure 2:**
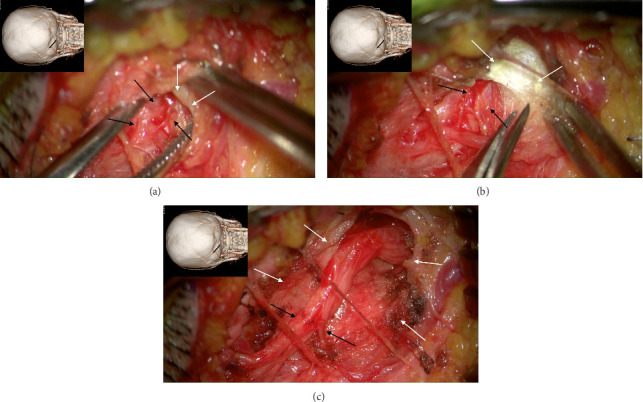
Intraoperative images showing the entrapment of the greater occipital nerve (GON) during the decompression. (a) Intraoperative photograph showing the GON (black arrows) located beneath the tendinous aponeurotic edge (white arrows) of the trapezius in the distal trapezius tunnel, lifted with forceps. The inset shows the direction of the image and the location of the incision. (b) Intraoperative photograph showing the tendon edge (black arrows) of the trapezius muscle being gradually dissected and cut. The white arrow represents the GON. (c) Intraoperative image after decompression of the left GON (black arrows) with division of the trapezial aponeurosis (white arrows). The GON region indicated by the black arrow represents the most severely compressed region.

The author's technique for decompression of the GON using an oblique, paramedian approach with a microscope has been previously described [12–20]. In summary, this approach begins with addressing the distal branch of the GON that traverses the trapezial tunnel. Under general anesthesia, a 3-cm long, paramedian oblique incision is made along the presumed course of the left GON. By identifying small visible branches of the GON in the subcutaneous tissue, the main branch of the GON is located, revealing compression against the tendinous aponeurotic edge of the trapezius muscle (Figure 2(a)). This site of entrapment is confirmed as the primary compression point for the GON. The tendinous aponeurotic edge is dissected, and the proximal course of the GON is circumferentially dissected (Figure 2(b)). The path is released by dissecting the semispinalis capitis muscle located beneath the trapezius until the point of GON emergence is reached (Figure 2(c)).

The effects of GON decompression were evident the day after surgery. At the 1-month follow-up, the patient reported approximately an 80% reduction in the constant pain and discomfort in various areas, including the left periorbital, preauricular, deep ear, temporo-occipital, and lateral neck regions. Notably, the intensity of the burning sensation in the left half of her tongue also decreased, specifically limited to the outer third (Figure 1(c)). The intensity of the pain was reduced by half, and she rarely experienced intermittent tingling pain in the left lower lip.

Three months postoperation, the patient reported the absence of pain in the left temporo-occipital and suboccipital areas, as well as in the periorbital, preauricular, and left jaw regions. This pain no longer affected her daily life. Although the extent of pain in the left tongue remained at the outer third, its intensity decreased by approximately 70% compared to preoperative levels. At her request, all medications were discontinued except for pregabalin 150 mg once daily, which she continued for residual burning pain in the tongue. One year after the surgery, the patient reported no headaches, neck pain, or facial pain. She experienced only mild burning pain in the left tongue, rated as one-third of the pain level prior to surgery (Figure 1(c)). There was no further change in the extent of pain in the left tongue, which remained confined to the lateral third. The pain was deemed tolerable with the use of pregabalin 75 mg once daily (3 out of 10 on the NRS-11).

## 3. Discussion

### 3.1. The GON: Major Nociceptive Afferent in Posterior Head and Neck

The GON originates from the medial branch of the dorsal rami of the second cervical nerve root, and it also receives a branch from the dorsal rami of the C3 root [28, 29]. It runs through the semispinalis capitis muscle, ascending and then continuing rostrolaterally beneath the trapezius muscle. After that, it passes through the tendinous aponeurotic sling located between the trapezius and sternocleidomastoideus muscles near their attachment to the superior nuchal line [28, 29]. This aperture is a common site of GON entrapment. It was named a trapezial tunnel [30, 31]. After piercing the aponeurotic sling of the trapezial tunnel, the GON widens as it continues toward the periphery, which differentiates it from other peripheral nerves [32]. This finding is considered relevant to GON entrapment because when the nerve widens, it becomes more susceptible to entrapment, particularly in the trapezius aponeurosis [14–20].

Structures located in the occipital and suboccipital regions, including vessels and the dura mater of the posterior fossa, deep paraspinal neck muscles, zygapophyseal joints, and ligaments, receive innervation from the upper cervical roots [6, 24, 33, 34]. These structures are commonly associated with neck and head pain [1, 5]. The nociceptive inflow from these suboccipital structures is mediated by small-diameter afferent fibers in the upper cervical roots. These fibers terminate in the dorsal horn of the cervical cord, which extends from the C2 segment up to the medullary dorsal horn [1, 5]. The major afferent input from the upper cervical root is carried through the C2 spinal root, which is represented peripherally by the GON [1, 5, 35]. Since the GON serves as the primary sensory afferent nerve through the C2 root, it directly transmits the nociceptive information to the C2 dorsal horn [1, 5].

### 3.2. Convergence of Trigeminal and Cervical Nociceptive Afferents and Sensitization of TBSNC

Trigeminal nociceptive afferents terminate in the caudal trigeminal nucleus (Sp5C) and the dorsal horn of the upper cervical cord up to C2 [8, 11, 26, 33]. Sp5C is referred to as the medullary dorsal horn due to its unique layered structure and similar morphological and functional organization to that of the spinal dorsal horn [6, 26]. The nociceptive neurons in the C2 dorsal horn receive primary afferents from the upper cervical roots, which are primarily represented by the GON [36]. It has been suggested that there is an anatomical overlap of trigeminal and high cervical afferents within the TCC, starting from the level of the Sp5C and extending to at least the C2 spinal cord [37]. Following the stimulation of nociceptive afferents, particularly C-fibers, a group of neurons in the TCC receives input from both the trigeminal and upper cervical areas. These neurons undergo neuroplastic changes, which resulted in an elevated level of central neuronal excitability [38].

Convergence and sensitization of secondary neurons in the TCC, located in the brain stem and upper spinal cord, provide a physiological explanation for the spread and referred pain observed between trigeminal and cervical areas. This means that pain originating from an affected tissue is perceived as originating from a different area [1, 2, 5]. Research has showed that Sp5C trigeminovascular neurons, which receive input from both the dura and periorbital area, not only increase their responses to inflammatory agents applied to dura but also become sensitized for several hours [8]. These sensitized Sp5C neurons have lower thresholds for both dural and periocular skin stimulation, and their dural and cutaneous receptive fields increase in size [8]. Based on these findings, it has been suggested that the cutaneous allodynia experienced by migraine patients is a result of central sensitization of Sp5C neurons following peripheral sensitization of meningeal nociceptors [39]. Stimulation of the GON leads to increased excitability of secondary nociceptive neurons that receive convergent input from supratentorial dura and the GON [1]. This demonstrates a direct functional connection between trigeminal and cervical afferents in the spinal dorsal horn [5].

In studies using animal models, chronic orofacial neuropathic pain has been observed when lesions occur in either the peripheral or central components of the trigeminal somatosensory system. These lesions can lead to changes in the TBSNC and associated behavioral changes [6, 27]. Various factors, such as injury to trigeminal nerve branches (e.g., the inferior alveolar nerve), administration of orofacial inflammatory drugs, or injection of algesic chemicals into orofacial tissues, can cause alterations in the trigeminal primary afferent nerve. This, in turn, can result in peripheral sensitization, a hyperexcitable state of the afferent nerve [6, 27]. It is important to note that changes in the excitability of trigeminal primary afferents following nerve injury or inflammation may extend beyond the damaged afferents. Adjacent intact afferents may undergo local sprouting, and there may be neuron-glial interactions involving the release of chemical mediators and changes in signaling mechanisms. These processes can lead to the spread of hypersensitivity in the TBSNC, upper cervical dorsal horn, and extraterritorial secondary hyperalgesia [6, 27, 40].

In addition, facial hypersensitivity may occur following spinal nerve injury in an animal pain model [27]. Unilateral injury to upper cervical spinal nerves or inflammation of tissues innervated by spinal nerves can lead to bilateral facial hypersensitivity and trigeminal central sensitization, which is associated with expanded neuronal receptive fields beyond their normal territory [41, 42]. These findings are consistent with previous clinical reports that nociceptive inputs to CNS from tissues supplied by spinal nerves can induce an extraterritorial sensory abnormality and spread of sensitivity (ETSS) to the trigeminal system [27]. ETSS between trigeminal and spinal nociceptive systems suggests that central sensitization and accompanying behavioral changes might occur in different body regions under chronic orofacial pain conditions such as temporomandibular disorders (TMDs) [27].

### 3.3. Entrapment of the GON and Referred Pain

Reports have indicated that chronic noxious input from GON entrapment can lead to sensitization and hypersensitivity of the secondary neurons in the TCC, resulting in referred pain in the distribution of the facial trigeminal nerve [12–20]. The clinical manifestations of GON entrapment–induced referred trigeminal pain are varied, with referral to not only in the ophthalmic (V1) region but also in the maxillary (V2) and mandibular (V3) regions. In some cases, this condition can even cause sensory changes and pain on one side of the face, along with occipital headaches [13]. Referred trigeminal pain from GON entrapment has also been associated with deep ear pain, which may occur years before the onset of occipital pain [16]. Furthermore, this referred pain can extend to the ipsilateral extremities, which are part of the spinal nociceptive area, indicating central sensitization [18]. In addition, there have been reports of extracephalic, generalized extension of referred trigeminal pain resulting from GON entrapment [19].

Two hypotheses have been proposed to explain the pathophysiology of extra-trigeminal extension of referred trigeminal pain from GON entrapment [19]. One hypothesis suggests that the third-order thalamic neurons may become hypersensitive [24, 25]. This hypothesis is supported by the fact that referred pain from GON entrapment can extend beyond the trigeminal nerve distribution of the face to the back and limbs. Therefore, not only the nociceptive second-order neurons in the brainstem but also central sensitization involving the third-order neurons may be involved in the pathophysiology. This phenomenon has already been demonstrated in migraine, a primary headache, where cutaneous allodynia can spread from the referred pain area on the ipsilateral head to the other side of the head and/or the forearms [24, 25]. Similarly, in pain disorders such as fibromyalgia, focal somatic pain may be associated with cutaneous allodynia (perception of pain evoked by innocuous stimuli) and hyperalgesia (sensitivity to noxious stimuli) that have spread beyond the original site of pain [43].

Another explanation for the extracephalic, generalized extension of referred pain involves more rostral Sp5C neurons and brainstem pain modulating structures and sensitization of TCC neurons located in the caudal Sp5C. Hypersensitivity of nociceptive processing pathways due to central sensitization in TBSNC can simultaneously lead to dysfunction of endogenous pain inhibitory pathways [26, 27]. There are important brainstem pathways of descending modulation (e.g., rostral ventromedial medulla [RVM] or subnucleus reticularis dorsalis [SRD] in the reticular formation) that receive trigeminal nociceptive inputs and send modulatory influences to areas of the spinal cord (e.g., spinal dorsal horn) involved in nociceptive transmission from the body [27, 44, 45]. Dysfunction of the endogenous pain inhibitory system in the brainstem has been observed in diseases such as fibromyalgia, TMD, and atypical facial pain. Central sensitization is considered the primary cause in these conditions [27, 34, 46, 47].

### 3.4. Referred Trigeminal Pain to the Tongue and Deep Ear in GON Entrapment

In this particular case, it has been confirmed that the patient experienced pain in half of the tongue and ipsilateral lip for a period of 20 years. However, this pain was significantly improved after undergoing GON decompression. It is important to note that the lingual branch innervates the anterior two-thirds of the tongue, while the mental branch innervates the lower lip. Both of these branches are part of the mandibular division (V3) of the trigeminal nerve. After careful examination, the patient's chronic pain in the tongue and lip was diagnosed as atypical facial pain, as no organic cause could be identified. According to the IASP classification, this type of pain is currently referred to as persistent idiopathic facial pain (PIFP) [48]. PIFP is defined as persistent facial and/or oral pain that occurs daily for more than 2 h a day, for a period of more than 3 months. This pain may present in different ways, but it is important to note that there is no accompanying clinical neurological deficit [48]. This could realistically be considered a provisional diagnosis for chronic tongue and lip pain of unknown origin. However, in order for it to meet the diagnostic criteria for PIFP, the pain must not be limited to a single peripheral nerve distribution and must be characterized as dull, aching, or nagging in nature [48]. However, the patient's pain was localized to the area supplied by the mandibular nerve (V3), and the characteristics of the pain did not align with the diagnostic criteria of PIFP. Another potential diagnosis to consider is burning mouth syndrome (BMS) [48]. However, in BMS, the pain is usually experienced bilaterally and is commonly felt at the tip of the tongue [48]. No systemic illness that could cause BMS was identified in the current case [48]. Therefore, it is challenging to accurately categorize referred trigeminal pain resulting from GON entrapment within the existing classification of headache and facial pain.

The immediate improvement of persistent pain in the left tongue and lip, which had been present for 20 years, GON decompression provides evidence that the pain in the mandibular division of the trigeminal nerve was actually referred pain caused by GON entrapment. It is interesting to note that there was a reorganization of the pain distribution, with the painful area of the tongue reducing from the left half to the lateral third. The remodeling of pain distribution and improvement of referred trigeminal pain by GON decompression suggest that this referred pain is associated with central pathophysiology, and the hypersensitivity of the secondary nociceptive neurons in the caudal trigeminal nucleus and dorsal horn of the high cervical cord is reversible. Therefore, in case of chronic persistent trigeminal pain that is difficult to explain, particularly perioral pain, accompanied by pain in the temporo-occipital and lateral neck regions, it is important to considered referred trigeminal pain caused by GON entrapment.

The current case showed that GON decompression improved the aching pain in the left eye, preauricular cheek, and deep inside the ear, along with temporo-occipital and lateral neck pain. It is evident that the periorbital pain, accompanied by temporo-occipital and lateral neck pain, which are typical symptoms of GON entrapment, are actually referred pain in the trigeminal nerve distribution's ophthalmic division (V1). Similarly, preauricular cheek pain is understood to be pain referred to the distribution of the mandibular division's (V3). Previous reports have also showed that pain referrals to the orofacial region due to GON entrapment can occur in both the ophthalmic (V1) region and the maxillary (V2) and mandibular (V3) regions [12–19].

Deep ear pain caused by GON entrapment has been reported previously [16]. Initially, this type of pain, which is not well localized, may be difficult to comprehend. However, it is important to note that, apart from the auricle, there are main sensory nerves that contribute to the sensory innervation of the external auditory canal and tympanic cavity [21, 22]. These are the auricular branch of the vagal nerve, chorda tympani of the facial nerve, tympanic branch of the glossopharyngeal nerve, and auriculotemporal nerve (V3) [21, 22]. The anterior and superior walls of the external acoustic meatus are innervated by the auriculotemporal branch of the mandibular nerve (V3). On the other hand, the posterior and inferior walls are innervated by the auricular branch of the vagal nerve [21]. The facial nerve may also contribute via its communication with the vagal nerve [21]. Deep ear pain can be understood rationally as referred pain in the area innervated by the auriculotemporal nerve. This nerve is a branch of the mandibular division (V3) of the trigeminal nerve, which innervates the anterior wall of the external auditory canal. The caudal trigeminal nucleus is where the nociceptive afferent information from the facial, glossopharyngeal, and vagal nerves, as well as the trigeminal afferent, terminate [23]. It is possible that chronic noxious stimulation of the GON entrapment may have led to central sensitization of the TBSNC and upper spinal cord. As a result, this could have caused referred pain not only in the trigeminal nerve but also in the inner ear's innervation by the glossopharyngeal and vagal nerves.

When trigeminal pain referred from GON entrapment becomes chronic, stubborn, and unresponsive to medical treatment over many years, patients who have undergone invasive interventions are often identified. These interventions should be considered in order to control the paroxysmal stabbing pain that is typical of trigeminal neuralgia [49]. The pain associated with classic trigeminal neuralgia is not felt in the tongue or deep ears. It is important to note that all neuroablative treatments for trigeminal neuralgia come with potential risks, including complications such as dysesthesia and deafferentation pain such as anesthesia dorolosa [49]. In this particular case, the patient underwent chemical rhizotomy using alcohol to treat trigeminal neuralgia. Fortunately, there were no sensory complications, but unfortunately, it did not effectively alleviate the referred pain.

## 4. Conclusion

Although previous reports have indicated that referred trigeminal pain due to GON entrapment can occur not only in the ophthalmic nerve (V1) but also in the V3 and V3 branches, in this particular case, the pain was experienced in the ipsilateral tongue. The tongue, an intraoral structure, receives sensory innervation from the lingual branch of the mandibular nerve (V3). In addition, referred pain was also felt in the ipsilateral lip, which receives sensory distribution from the inferior alveolar branch of the mandibular nerve (V3). It is worth noting that this tongue and lip pain began long before the full onset of occipital pain due to GON entrapment and has persisted for 20 years. This referred trigeminal pain can be attributed to the sensitization and hypersensitivity of the secondary nociceptive neurons in the caudal trigeminal nucleus. This occurs because both trigeminal and occipital nociceptive afferents converge on the MDC and TCC. In fact, after GON decompression, the pain experienced significantly improved, with a notable reduction in the extent of tongue pain. This suggests a central pathophysiology.

## Data Availability

Research data are not shared.
